# G9a controls pluripotent-like identity and tumor-initiating function in human colorectal cancer

**DOI:** 10.1038/s41388-020-01591-7

**Published:** 2020-12-15

**Authors:** Christopher J. Bergin, Aïcha Zouggar, Joshua R. Haebe, Angelique N. Masibag, François M. Desrochers, Simon Y. Reilley, Gautam Agrawal, Yannick D. Benoit

**Affiliations:** Department of Cellular and Molecular Medicine, University of Ottawa, Ottawa, ON K1H 8M5 Canada

**Keywords:** Cancer stem cells, Histone post-translational modifications, Prognostic markers

## Abstract

Colorectal tumors are hierarchically organized and governed by populations of self-renewing cancer stem cells, representing one of the deadliest types of cancers worldwide. Emergence of cancer stemness phenotype depends on epigenetic reprogramming, associated with profound transcriptional changes. As described for pluripotent reprogramming, epigenetic modifiers play a key role in cancer stem cells by establishing embryonic stem-like transcriptional programs, thus impacting the balance between self-renewal and differentiation. We identified overexpression of histone methyltransferase G9a as a risk factor for colorectal cancer, associated with shorter relapse-free survival. Moreover, using human transformed pluripotent cells as a surrogate model for cancer stem cells, we observed that G9a activity is essential for the maintenance of embryonic-like transcriptional signature promoting self-renewal, tumorigenicity, and undifferentiated state. Such a role was also applicable to colorectal cancer, where inhibitors of G9a histone methyltransferase function induced intestinal differentiation while restricting tumor-initiating activity in patient-derived colorectal tumor samples. Finally, by integrating transcriptome profiling with G9a/H3K9me2 loci co-occupancy, we identified the canonical Wnt pathway, epithelial-to-mesenchyme transition, and extracellular matrix organization as potential targets of such a chromatin regulation mechanism in colorectal cancer stem cells. Overall, our findings provide novel insights on the role of G9a as a driver of cancer stem cell phenotype, promoting self-renewal, tumorigenicity, and undifferentiated state.

## Introduction

Colorectal cancer (CRC) is accountable for over 860,000 deaths per year worldwide [1]. Advanced stages and recurrent/metastatic CRCs in particular show poor 5-year survival rates [2]. Thus, a better understanding of mechanisms fostering disease progression and dissemination is critical to develop novel, improved therapeutic strategies lowering CRC-associated mortality. Cellular heterogeneity within colorectal tumors is hierarchically organized and governed by populations of self-renewing cancer stem cells (CSC) [3]. CSCs were shown to evade standard genotoxic and antimetabolite-based therapies, making them particularly difficult to eradicate [4]. Moreover, CSCs possess tumor-initiation capacity, thereby promoting CRC relapse and metastasis [5].

Compelling evidence indicates that cancer stemness is closely related to pluripotency gene expression signatures in human tumors and such dysfunctional embryonic-like transcriptional programs strongly correlate with aggressiveness, metastasis, and fatality [6–8]. This was specifically reported in epithelial neoplasms such as CRC and relates to the undifferentiated phenotype associated with CSCs [7, 8]. As demonstrated in the context of development and pluripotent reprogramming, epigenetic modifiers play a key role in establishing embryonic stem (ES) transcriptional programs and mediating the balance between self-renewal and differentiation [9, 10]. Similarly, alterations in chromatin regulation mechanisms are implicated in the development and maintenance of CSCs and define intratumoral heterogeneity via epigenetic reprogramming [11–13]. Such a concept is applicable to CRC, where Polycomb group proteins were demonstrated as essential to maintain neoplastic stemness features, in part by regulating pluripotency networks [14, 15]. Histone H3 lysine-9 (H3K9) methylation is among other epigenetic determinants that recently received significant attention regarding CSC activity. Specifically, the histone methyltransferase (HMTase) G9a, which catalyzes H3K9 mono and di-methylation (H3K9me1/2), was linked to self-renewal and tumor initiation in several neoplasms including leukemia, melanoma, lung tumors, as well as head and neck carcinomas [16–19]. Although previous observations supported a role for G9a and H3K9me2 deposition in CRC by influencing proliferation and the chemotherapeutic response [20, 21], little is known about the functional implication of H3K9me2-regulated networks in the maintenance of CSCs [22].

Here, we investigated the impact of inhibiting G9a expression and H3K9me2 deposition on pluripotent-like neoplastic stemness programs. Using human primary CRC tissues and a robust transformed pluripotent stem cell model, we established a fundamental relationship between G9a/H3K9me2 and CSC-related transcriptional networks mediating self-renewal and differentiation. Moreover, pharmacological inhibition of H3K9me2 deposition significantly reduced tumor-initiating activity in patient-derived CRC stem cells, emphasizing the importance of G9a as a therapeutic target to suppress CSC activity [22].

## Results

**Fig. 1:**
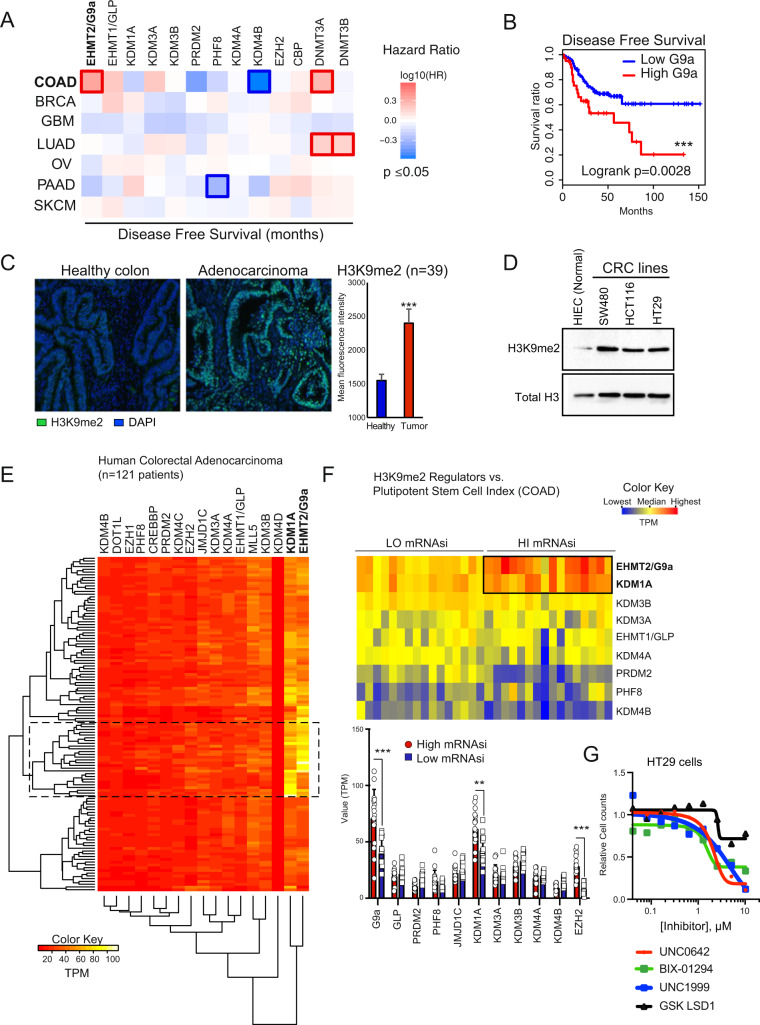
G9a expression is associated with pluripotent-like transcriptional program and poor prognosis in human CRC. **A** Survival map representing prognostic impact of genes encoding key chromatin regulators, based on patient’s disease/relapse-free survival and expression levels. EHMT2/G9a expression levels display a negative impact on disease-free survival in colon adenocarcinoma patients (TCGA COAD). The heat map shows the hazard ratios in log10 scale for tested genes. Red/blue frames indicate significant unfavorable/favorable risk in prognostic analyses (*p* value ≤ 0.05). **B** Kaplan–Meier disease-free survival analysis of patients presenting high (top 15%, TPM + 1) and low (bottom 85%, TPM + 1) G9a expression levels in primary CRC tumors (TCGA COAD, *n* = 275, Logrank *p* = 0.0028: ***). **C** Immunofluorescence detection of H3K9me2 in human colon adenocarcinoma sections (*n* = 39 patients) vs. normal colonic mucosa. Mean fluorescence intensity was quantified using ImageJ (*p* = 0.0007, ***). **D** Western analysis of H3K9me2 levels in whole-cell lysates from human normal intestine epithelial crypt cells (HIEC) vs. CRC cell lines SW480, HCT116, and HT29. Total histone H3 was used as loading control. **E** RNA-seq profiling of key chromatin regulators in human colon adenocarcinoma (TCGA COAD, *n* = 121). Values are expressed as TPM. **F** Expression of main H3K9me2 regulators in COAD samples displaying “high” (>0.6, *n* = 16) and “low” (<0.3, *n* = 16) mRNA expression-based pluripotent stem cell indices (mRNAsi). TPM values were compared between HI and LO mRNAsi groups for each H3K9me2 regulator (***:*p* ≤ 0.0001; **:*p* = 0.0009). **G** Dose-response experiment assessing the impact of G9a (BIX-01294 and UNC0642), EZH2 (UNC1999), and LSD1 (GSK-LSD1) inhibition on HT29 cell growth (*n* = 4).

We sought to determine which epigenetic pathways are prominently associated with therapeutic failure and relapse in CRC. Relapse-free survival (RFS) analysis in various cohorts determined the risk factors associated with 13 key chromatin regulators across different cancer types. We identified EHMT2/G9a (Hazard ratio (HR): 2.2, *p* = 0.0036) and DNMT3A (HR: 1.9, *p* = 0.047) overexpression to be significantly associated with shorter RFS in colon adenocarcinomas (COAD) (Fig. 1A, B, Fig. S1A). In contrast, high expression of KDM4B —an H3K9-specific demethylase—was linked to enhanced RFS prognosis in colon tumors (HR: 0.27, *p* = 0.028) (Fig. 1A, Fig. S1A). The expression of G9a binding partner EHMT1/GLP, essential for H3K9 mono and di-methylation, was not associated to RFS in CRC patients (Fig. S1A). Global H3K9me2, mainly catalyzed by G9a, was significantly increased in human COAD vs. normal colonic mucosa (Fig. 1C), as well as in three human CRC lines compared to normal intestinal progenitor HIEC cells (Fig. 1D). Clustering analysis of chromatin modifier expression across 121 COAD patients indicated that EHMT2/G9a and KDM1A/LSD1 mRNAs are particularly abundant in a specific subset of patients (Fig. 1E, Table-S1). Akin to KDM4B, LSD1 lysine demethylase activity impacts H3K9 modification status [23]. Compared to healthy colon, G9a and LSD1 transcript expression is significantly higher in CRC vs. other H3K9me2 regulators (Fig. S1B). Considering the importance of G9a in oncogenic self-renewal [17] and developmental cell fate regulation [10], we scrutinized the potential relationship of G9a with pluripotent-like transcriptional signature and tumor stemness [6, 7]. Using a one-class logistic regression machine-learning approach, a transcriptional stem cell index (mRNAsi) was determined for 121 individual CRC tumors based on the gene expression signature of human pluripotent cells [6]. CRC samples were categorized into “High mRNAsi” (≥0.6) and “Low mRNAsi” (≤0.3) groups (Table-S1). Interestingly, 14 out of 16 samples falling into the High mRNAsi group (≥0.6, *n* = 16) were found within the cluster of high G9a/LSD1-expressing patient samples (Fig. 1E, F, and Table-S2). This coincides with the elevated transcript levels of these chromatin modifiers in the highly tumorigenic CRC lines HCT116 and HT29, both recognized to contain large subpopulations of CSC-like cells, compared to normal HIEC and less-tumorigenic SW480 cells (Fig. S1C) [24, 25]. We also observed that EZH2-overexpressing tumors were correlated to high mRNAsi (Fig. 1F). This supports previous findings identifying EZH2 as another chromatin regulator maintaining colorectal cancer stem cell (CCSC) functions [15]. When using pharmacological inhibitors of G9a and LSD1 on CRC cells, we observed lower EC_50_ values for BIX-01294 and UNC0642 (G9a inh.: 1.65μM and 2.58μM respectively) compared to LSD1 inhibition (GSK-LSD1: 22.1μM) (Fig. 1G). G9a inhibition displayed comparable effects on cell growth vs. EZH2 inhibition (UNC1999, EC_50_: 1.59 μM) used as a positive control in this experiment. Thus, G9a expression could contribute to the elevated risk of relapse by driving tumor stemness and establishing pluripotent-like transcriptional programs in human CRC.

**Fig. 2:**
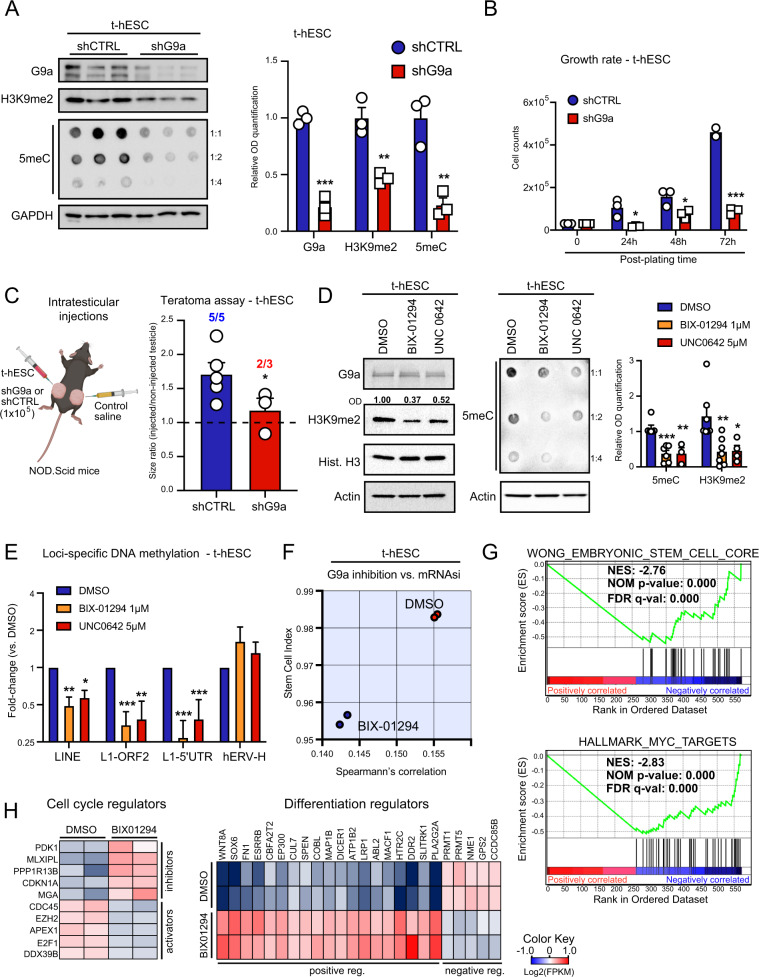
Suppression of G9a activity reduces pluripotent identity and tumor growth capacity in transformed human embryonic stem cells (t-hESCs). **A** Western blot analysis of G9a, H3K9me2, and dot blot analysis of 5-methylcytosine (5meC: methylated DNA) levels in G9a knockdown t-hESCs vs. scramble control shRNA (shCTRL). GAPDH was used as loading control. Relative OD signal quantification vs. GAPDH intensity are presented (*n* = 3, ***: *p* = 0.0001; **: *p* ≤ 0.0057). **B** Growth rate assessment of shG9a vs. shCTRL t-hESC cultures. Cell counts were acquired at 24 h (*: *p* = 0.021), 48 h (*: *p* = 0.034), and 72 h (***: *p* = 0.0003) post-seeding (30 K cells/well, *n* = 3). **C** Testicular teratoma assay using shCTRL (*n* = 5) and shG9a (*n* = 3) t-hESCs over 14 days. 1 × 10^5^ cells (left testicle) or saline (right testicle) were injected in each mouse. Tumor formation frequencies and tumor/control size ratios were presented for both groups (*: *p* = 0.043). **D** Western blot analysis of H3K9me2 and dot blot analysis of 5 meC levels upon pharmacological inhibition of G9a HMTase activity (BIX-01294: 1 μM, UNC0642: 5 μM, 48 h) in t-hESCs (H3K9me2: *n* ≥ 4, **: *p* = 0.0067; *: *p* = 0.031; 5 meC: *n* ≥ 3, ***: *p* = 0.00057; **: *p* = 0.0083). **E** Methylated DNA immunoprecipitation (MeDIP) assay showing variations of methylation levels in t-hESCs LINE transposable elements upon BIX-01294 (1 μM) and UNC0642 (5 μM) treatments, relative to DMSO controls (total LINEs: *n* = 3, **: 0.0082; *: *p* = 0.013; LINE-1 ORF2: *n* = 5, ***: *p* = 0.0004; **: *p* = 0.0072; LINE-1 5′-UTR: *n* = 6, ***: *p* ≤ 0.00089). Methylation of endogenous retroviral elements hERV-H was not affected by G9a inhibition (*n* = 6). **F** mRNAsi from RNA-seq transcriptome profiling was determined for BIX-01294 (1 μM) and DMSO treated t-hESCs. **G** GSEA revealed negative correlations between significant transcriptional modulations induced by BIX-01294 in t-hESCs (vs. DMSO, *p* < 0.05) and genes upregulated in embryonic stem cell, as well as genes induced by MYC. **H** Heat maps representing expression of significantly modulated genes (*p* < 0.05) associated with cell cycle (Negative Reg.: GO:0045786, Positive Reg.: GO:0045787) and differentiation regulation (Positive Reg./Stem Cell Diff.: GO:0045597, GO:0048863, Negative Reg.: GO:0045596) in BIX-01294-treated t-hESCs vs. DMSO controls.

Next, we studied the role of G9a in the context of neoplastic stemness using a transformed variant of H9 human ES cells (t-hESC), as a surrogate model for CSC in adherent cultures [26, 27]. t-hESCs faithfully recapitulate the main functional, transcriptional, and epigenetic characteristics of CSCs in culture and in vivo [24, 26–28]. Knockdown of G9a yielded significant reductions of H3K9me2 and global DNA methylation (5-methylcytosine: 5meC) in t-hESCs (Fig. 2A, and Fig. S2A). Notably, G9a knockdown reduced the growth rate of t-hESCs (Fig. 2B) and induced morphological aspects of non-transformed, parental H9 cells (Fig. S2B). To evaluate the impact of G9a knockdown on t-hESC tumorigenicity, in vivo teratoma assays were performed and the size of testicular tumors were monitored 14 days post-inoculation. We observed significantly reduced tumor growth in G9a knockdown injected animals vs. control group (scrambled shRNA) (Fig. 2C). Akin to G9a knockdown, the use of two G9a inhibitors, BIX-01294 and UNC0642 reduced the levels of H3K9me2 and global DNA methylation in t-hESCs (Fig. 2D). G9a inhibition exhibited no effects on H3K27 mono-methylation and EZH2-catalyzed H3K27me3 (Fig. S2C).

Thus, we conducted methylated DNA immunoprecipitation (MeDIP) experiments to further study the impact of G9a inhibition on CSC methylome. Given the drastic changes in 5meC levels observed in t-hESCs upon G9a inhibition (Fig. 2D), we focused our analysis on abundant/repeated retrotransposable elements, such as Long Interspersed Elements (LINE) and endogenous retroviruses (hERVs) [29]. Inhibition of retrotransposon transcriptional activity is highly dependent on DNA methylation and is critical to preserve pluripotent stem cell identity [29]. MeDIP assays revealed that G9a inhibition using BIX-01294 and UNC0642 significantly reduced DNA methylation in ORF2 and 5′-UTR regions of LINE1 elements (Fig. 2E, and Fig. S2D, E). This was accompanied by significant increases in LINE1-encoded transcripts (Fig. S2F), supporting reactivation of such retrotransposable elements in response to hypomethylation. No changes in DNA methylation were observed in human ERV-H elements (hERV-H) upon G9a inhibition vs. DMSO controls (Fig. 2E). MeDIP values expressed as percentage to input, along with transcriptional analysis suggest that hERV-H elements, which are required to maintain pluripotency, would exhibit low DNA methylation levels independently of G9a activation state (Fig. S2E, F) [30]. Furthermore, we used transcriptome profiling data from control and BIX-treated t-hESC to determine the impact of G9a inhibition on gene networks associated with neoplastic stemness. Transcriptomic data were used to assess changes in t-hESC mRNAsi upon G9a inhibition. Although the expression of core pluripotency factors was not altered upon G9a inhibition, we observed a downshift in t-hESCs mRNAsi score in response to BIX treatments (Fig. 2F, Fig. S2G, H). Gene signature enrichment analysis (GSEA) performed on genes significantly modulated by BIX in t-hESCs revealed negative correlation patterns with an ES cell transcriptional signature and targets upregulated by the proto-oncogene c-Myc (Fig. 2G, Table-S3) [7]. In addition, GSEAs suggested the restoration of p53 target expression upon G9a inhibition in t-hESCs (Fig. S2I) [31]. Accordingly, the inhibition of H3K9me2 deposition in t-hESCs was marked by a significant upregulation of genes restricting proliferation, such as p21 (CDKN1A), and the repression of genes associated with cell cycle progression (e.g., CDC45) (Fig. 2H, and Fig. S2J). Consistent with its impact on mRNAsi, G9a inhibition also induced the expression of genes regulating cell fate and differentiation (Fig. 2H, and Fig. S2J). Thus, our observations regarding the role of G9a in the context of neoplastic pluripotency are consistent with risks associated with its overexpression in CRCs and support the relevance of such an epigenetic mechanism as a potential therapeutic axis.

**Fig. 3:**
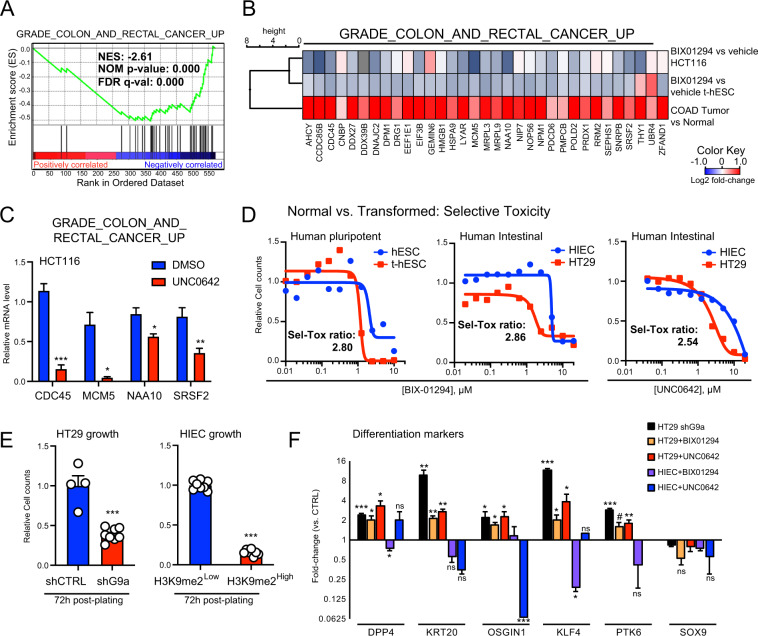
G9a inhibition is selectively inducing intestinal differentiation program in human CRC cells. **A** GSEA showing a negative correlation between genes significantly modulated by BIX-01294 in t-hESCs (vs. DMSO, *p* < 0.05) and genes upregulated in human CRCs. **B** Hierarchal clustering analysis of BIX-01294-treated t-hESC and HCT116 cells, as well as COAD samples (vs. normal tissues) based on CRC upregulated genes. **C** Candidate-based validation of Grade CRC upregulated (M16740) genes upon G9a inhibition using UNC0642 (2.5 μM, 48 h) in HCT116 cells (*n* = 3, ***: *p* = 0.0006; **: *p* = 0.0045; *: *p* ≤ 0.028). **D** Dose-response curves assessing selective toxicity (Sel-Tox) of G9a inhibition (BIX-01294) in t-hESCs vs. normal human ES cells (hESC), as well as cancer stem-like HT29 vs. normal HIEC cells (BIX-01294 and UNC0642). Sel-Tox ratios were determined as follow: EC_50_Normal/EC_50_Cancer (*n* = 3). **E** Growth rate assessment of G9a knockdown (shG9a, *n* = 8) vs. non-silencing control (shCTRL, *n* = 4) HT29 cells, and H3K9me2^High^ (*n* = 8) vs. H3K9me2^Low^ (*n* = 8) HIEC cells at 72 h post-seeding (***: *p* ≤ 0.0001). **F** Quantitative PCR assessment of intestinal differentiation markers expression in G9a knockdown, BIX-01294 (1 μM, 48 h) and UNC0642-treated (2.5 μM, 48 h) HT29 cells, as well as BIX-01294 (1 μM, 48 h) and UNC0642-treated (2.5 μM, 48 h) HIEC cells (*n* ≥ 3, ***: *p* ≤ 0.0008; **: *p* ≤ 0.008; *: *p* ≤ 0.047; #: *p* = 0.052). Results are expressed as mRNA fold-change vs. respective controls (shCTRL and DMSO). GAPDH was used as a reference gene.

Similar to our approach in the transformed pluripotent model, we investigated the impact of G9a inhibition on the regulation of oncogenic transcriptional networks in CRC. Notably, GSEA performed on BIX-modulated genes in t-hESCs demonstrated a striking inverse correlation with a set of genes overexpressed in CRCs (Fig. 3A) [32]. Hierarchal gene clustering analysis on such a CRC-specific gene signature highlighted similarities in transcriptional responses to G9a inhibition between t-hESCs and HCT116 CRC cells (vs. vehicle controls) (Fig. 3B). This was further validated by a candidate-based qPCR approach in UNC0642-treated HCT116 (Fig. 3C). Moreover, a majority of genes associated with the ES cell transcriptional program and downregulated by BIX in t-hESC (Fig. 2F) were also repressed by BIX and UNC0642 treatments in the CSC-enriched cell line HCT116 (Fig. S3A, B) [25]. A similar relationship was observed for p53 target genes, when comparing the transcriptional response to G9a inhibition in t-hESCs (Fig. 2F) vs. HCT116 cells (Fig. S3A, B). Considering the important shifts in transcriptional programs upon G9a inhibition, we tested the impact of G9a inhibition in CSC-like (t-hESCs and HT29 cells) vs. related normal models (H9 hESCs and intestinal progenitor HIEC cells). Akin to t-hESCs, BIX and UNC0642 substantially reduced H3K9me2 deposition in HT29 cells (Fig. S3C). In all cases, pharmacological inhibition of G9a led to selective toxicity toward neoplastic tissues (EC_50_ normal/EC_50_ cancer) (Fig. 3D). Accordingly, modulating H3K9me2 deposition by either knocking down G9a (decrease) or inhibiting lysine demethylase KDM4B (increase) had specific impacts on cell growth (Fig. 3E and Fig. S3C–F). While G9a knockdown impaired growth in CSC-like HT29 cells, inhibition of KDM4B using the small molecule NCGC00244536 had no effects on CRC cells (<10 μM), potentially due to a plateau in H3K9me2 deposition (Fig. S3D, E). However, when normal HIEC cells were treated with KDM4B inhibitor (48 h), we observed an increase in H3K9me2 deposition and a ~40% decrease in cell counts (Fig. S3D, E). We let surviving NCGC-treated HIEC cells (H3K9me2^High^) to recover for 24 h and subsequently assessed whether increased H3K9me2 deposition can modify normal intestinal progenitor growth rate vs. controls (H3K9me2^Low^). We observed that H3K9me2^High^ HIEC retained substantially slower growth rates compared to H3K9me2^Low^ cells at 72 h post-plating (Fig. 3E, Fig. S3D). We also observed that both, G9a knockdown and HMTase inhibition induced intestinal cell differentiation in HT29 cells based on markers such as KRT20, DPP4, KLF4, PTK6, and OSGIN1 (Fig. 3F), while G9a inhibition did not promote intestinal differentiation program in normal HIEC cells (Fig. 3F). Altogether, our observations in CRC/intestinal models confirm a key role for G9a in maintaining pro-oncogenic transcriptional programs responsible for compromising differentiation and promoting neoplastic stemness in colorectal tumors.

**Fig. 4:**
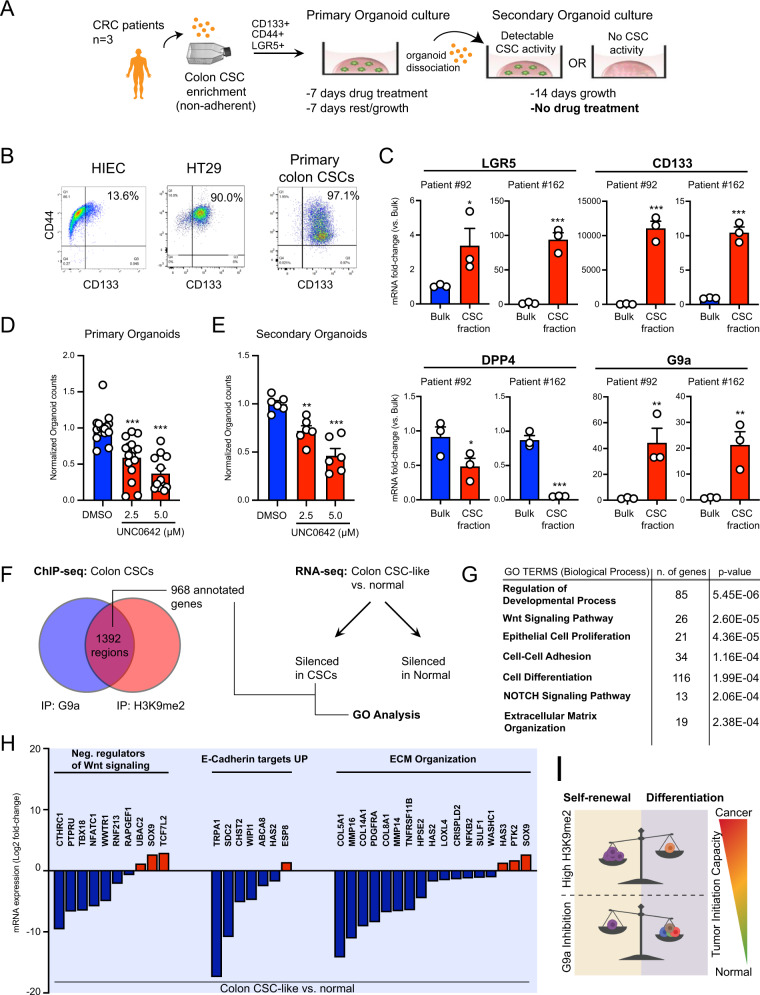
Pharmacological inhibition of G9a HMTase activity is reducing tumor-initiating capacity in primary CRC patient samples. **A** Schematic representation of serial organoid formation assay using primary human CRC tissues. Cancer stem cell (CSC) fraction is enriched in non-adherent spheroid cultures. **B** Flow cytometry profiling of patient-derived spheroid cultures for CCSC markers CD133 and CD44. Normal progenitor cells (HIEC) and CCSC-like HT29 were presented as controls. **C** Quantitative PCR assessment of CCSC markers LGR5, CD44, and CD113, as well as G9a expression in bulk primary organoids vs. CSC-enriched fractions (spheroid cultures) (Two patients tested, *n* = 3 per patient, ***: *p* ≤ 0.0004; **: *p* ≤ 0.0093; *: *p* ≤ 0.041). **D** Primary organoid formation frequency observed upon UNC0642 treatments (2.5 μM and 5 μM, 7 days). Organoid counts were normalized vs. DMSO controls (three patients, *n* ≥ 12, ***: *p* < 0.0001). **E** Organoid formation frequencies observed in secondary plating assays, for DMSO, UNC0642 2.5 μM and UNC0642 5 μM groups. Organoid counts were normalized vs. DMSO controls (three patients, *n* = 6, ***: *p* < 0.0001; **: *p* = 0.005). **F** Integrative analysis using ChIP-seq and RNA-seq transcriptome profiling to identify G9a and H3K9me2 co-enriched annotated genes in primary human CCSCs (GSE82131) that are silenced in HT29 (CCSC-like) vs. normal progenitors (HIEC) (*p* < 0.05). **G** Gene ontology analysis of G9a/H3K9me2 co-enriched genes and silenced in CCSC (vs. normal) reveals the implication of key pathways linked to tumorigenicity. **H** Diagram illustrating relative mRNA expression of G9a/H3K9me2 co-enriched genes in human CCSCs (vs. HIEC, *p* < 0.05) across key functional categories (GO:0030178, M18757, GO:0031012). **I** Recapitulative schematic highlighting the impact of G9a inhibition on the loss of pluripotent-like gene expression patterns and the onset of differentiation transcriptional programs in human CRC-initiating cells.

Subsequently, we explored the functional role of G9a HMTase activity in CCSC populations from patient-derived tumor samples (Fig. S4A). Using a serial organoid formation assay, we tested the potential of G9a inhibition to affect the tumor-initiating capacity of primary CCSCs (Fig. 4A). CCSC populations were enriched through formation spheroids in suspension, as marked by the large proportion of CD133^+^/CD44^+^ cells, comparable to the CSC-like HT29 cell line (Fig. 4B) [3, 24]. This was supported by the enrichment of LGR5 expression in patient-derived spheroids vs. bulk 3D organoids resulting from single-cell seeding (Fig. 4C) [3, 5]. By opposition to spheroids, 3D organoids were described as mini tumors maintaining primary patient tumor heterogeneity [15]. Interestingly, we observed reduced levels of the differentiation marker DPP4, along with a clear enrichment of G9a expression in patient-derived spheroids vs. bulk organoids (Fig. 4C). G9a inhibition in a primary series of patient-derived 3D organoids resulted in a significant decrease of organoid size and frequency compared to vehicle-treated groups (Fig. 4D and Fig. S4B, C). Residual primary organoids were dissociated and re-seeded in a secondary series. Importantly, no further drug treatments were performed on this secondary series of patient-derived 3D organoids, enabling bona fide assessment of persisting tumor-initiating cell populations in samples previously treated with a G9a inhibitor (vs. vehicle control) (Fig. 4A). Thus, BIX and UNC0642-treated primary organoids had lower tumor-initiating capacity when plated in a secondary assay (Fig. 4E, and Fig. S4C, D). Induction of H3K9me2 deposition in HIEC cells was insufficient to confer them with organoid formation capacity (Fig. S4C). Mechanistically, we searched for potential pathways involved in G9a-mediated maintenance of CSCs in CRC patient samples. Analysis of chromatin immunoprecipitation (ChIP-seq) data set revealed that G9a and H3K9me2 are significantly co-enriched at 1392 genomic regions (1 kb) in patient-derived CCSCs (Fig. 4F, Fig. S4E, and Table-S4) [18]. A majority of the 968 annotated co-enriched sites were mapped in introns (422) and distal intergenic regions (680) (Fig. 4F, Fig. S4F, and Table-S4). This is consistent with our MeDIP observations (Fig. 2E, and Fig. S2E), considering that LINE1 elements are observed at a high frequency within intronic and intergenic space [33]. Thus, we performed an integrative analysis of transcriptionally silenced genes in CSC-like HT29 cells vs. normal HIECs (RNA-seq, Fig. S4G) that are simultaneously enriched/co-occupied with G9a and H3K9me2 in patient-derived CCSCs (ChIP-seq) (Fig. 4F). We identified a list of 183 annotated entities presenting those criteria, which we used to perform gene ontology analysis (Table-S5 and S6). Genes putatively silenced by G9a in CCSCs were associated with processes such as regulation of development and cell differentiation (Fig. 4G, Fig. S4H, Table-S7). Moreover, multiple genes involved in negative regulation of canonical Wnt signaling (GO:0030178), repression of epithelium-to-mesenchyme transition (Onder E-cadherin targets up) [34], and extracellular matrix organization (GO:0031012) were silenced in CRC (vs. normal) and co-enriched with G9a and H3K9me2 in patient-derived CCSCs (Fig. 4H, Table-S7 and S8). Taken together, our data on primary CRC patient samples confirm G9a as a critical epigenetic factor maintaining CSC activity in human CRCs (Fig. 4I).

## Discussion

Herein we established a fundamental role for G9a/H3K9me2 as a regulator of pluripotency networks in CCSCs. Indeed, G9a expression levels significantly associate with poor clinical outcomes, but also correlate with pluripotent transcriptional signature, which was recently demonstrated as a robust indicator of tumor aggressiveness and neoplastic stem cell phenotype [6]. Moreover, pharmacological inhibition of G9a is effective to stimulate differentiation and to restrict self-renewal and tumorigenicity in CCSCs. As for previous studies using the same class of small molecule inhibitors (dual G9a/GLP inhibitors) [17–20], we generalized our findings to G9a functions, since (1) knockdown experiments recapitulated chemical treatments and (2) no impact on RFS was observed for GLP overexpression in CRC patients. Nevertheless, we cannot exclude the possibility that GLP modulation alone would show effects on CCSC biology. Although previous reports highlighted G9a-mediated H3K9me2 deposition as a driver of CSC biology in other cancers [17, 19], a better understanding of its function regulating CCSC epigenetic signature was necessary. Hence, our integrative multi-omics approach identified Wnt/β-catenin, extracellular matrix organization, and epithelium-to-mesenchyme transition as key networks regulated by G9a HMTase activity. These networks represent hallmarks of neoplastic self-renewal and de-differentiation (Fig. 4.I) [16, 18, 24, 35, 36]. Collectively, our data supports recent findings connecting G9a expression to cell proliferation and canonical Wnt activity in CRC [18, 20, 22], as well as previous demonstration that G9a expression enhances in vivo tumorigenicity and resistance to ionizing radiation in HT29 cells [37]. Beyond direct impacts on CSC biology, our results exposed the cancer-selective nature of G9a inhibition, when comparing growth and differentiation responses in neoplastic vs. normal tissues. This aligns with a rising trend suggesting G9a as an attractive therapeutic target, which would exhibit limited toxicity to healthy tissues [18–20, 22]. Our study also demonstrates that chemical induction of H3K9me2 deposition in normal intestinal progenitor cells is not sufficient to solely drive the acquisition of oncogenic characteristics such as enhanced growth and tumor initiation capacity.

Human pluripotent cells were shown useful to study cancer stemness [6–8, 28] and were used to identify and characterize thioridazine and CWP232228 as pharmacological agents suppressing CSC activity in human leukemia [24, 26]. In addition, the ES-like transcriptional signature in somatic tissues is recognized to elicit oncogenic self-renewal [7]. In such a context, c-Myc alone was specifically shown as sufficient to establish pluripotent-like programs, increasing tumor-initiating cell fractions by 150-fold [7]. G9a HMTase activity was extensively linked to early embryonic stages and regulation of pluripotent gene networks [22, 38, 39]. However, G9a inhibition in t-hESCs had no effects on pluripotency factor expression, including c-Myc. Still, we observed a robust inhibition of c-Myc targets when H3K9me2-deposition was inhibited, which strengthen the concept that G9a plays a key role in the establishment of an oncogenic pluripotent gene signature in CSCs.

Our survival analysis identified G9a overexpression as a risk factor for relapse in CRC, supporting H3K9me2 enrichment as a biomarker of the CRC epigenetic signature [40, 41]. DNMT3A overexpression also correlated with a higher risk for relapse in colon cancer. G9a is a known stabilizing factor promoting the recruitment of DNMT3A to imprinted loci in ES cells, independent of its HMTase activity [42]. G9a HMTase activity was linked to cancer stemness in lung carcinoma by maintaining oncogenic DNA methylation patterns via interaction with DNMT1, but not DNMT3A [19]. Both, knockdown and chemical inhibition of G9a induced a decrease of DNA methylation levels in t-hESCs. Further investigations would be necessary to determine the nature of the interactions taking place between G9a and DNA methylation machinery in CSCs. Yet, we identified LINE1 retrotransposable elements as a target of genomic hypomethylation induced by G9a inhibition in CSCs. Transcriptional activation of LINE1 elements, and anti-tumorigenic effects observed following G9a inhibition are consistent with a previous study showing that the reactivation of endogenous retro-elements can induce viral mimicry and block tumor-initiating functions in CRC patient samples [43]. Interestingly, our conclusions on G9a activity and its relationship with pluripotency networks in CRC show similarities with another recent study relating EZH2 activity to hedgehog signaling, core pluripotency factors, and maintenance of tumor-initiating functions in CCSCs [15]. An interplay between G9a and EZH2-dependent chromatin patterning was also documented, where G9a is involved in H3K27 mono-methylation, serving as a precursor to EZH2 recruitment and transcriptional silencing [39]. However, our data demonstrate that pharmacological inhibition of G9a does not influence H3K27 methylation status in the short term, raising doubts on the existence of an epigenetic regulatory hub simultaneously involving G9a and EZH2 in CCSC populations. Accordingly, our study demonstrates that suppression of G9a activity, alone, can inhibit tumor-initiating capacity in primary CRC samples. To date, several small molecule inhibitors of G9a were reported (e.g., BIX, UNC0642, A-366, CM-272), showing an ability to block H3K9me2 deposition via direct interaction with the enzyme [44]. However, we noted a paucity of in vivo investigations and lack of clinical trials using these compounds, potentially due to poor pharmacokinetics parameters. Despite such in vivo hurdles, additional work on alternative ways to regulate G9a expression or activity in cancer is still critical to develop novel, effective approaches targeting G9a for future therapies against CSCs.

## Materials and methods

(*see* Extended Materials and methods*section for details*).

### Tissue culture and reagents

Cell lines were obtained and cultured per recommendations from ATCC. Primary colorectal tumor tissues were obtained with patient consent, as approved by the University Health Network Research Ethics Board and from Celprogen Inc. (#36112-39P, Torrance, CA). Small molecule inhibitors were purchased from Tocris Bioscience.

### Immunofluorescence

Human colon carcinoma tissue microarray sections, including normal colon tissues (US Biomax, #CO486) and fixed cells were stained using anti-H3K9me2 primary antibody (Table-S9).

### Western blotting

Whole-cell extracts were migrated on polyacrylamide gels and transferred onto nitrocellulose membranes. For dot blot experiments, whole-cell extracts were directly spotted onto membranes. Primary antibodies used are described in Table-S9.

### In vivo *teratoma assays*

t-hESCs were injected intratesticularly into male NOD/SCID mice, according to the McMaster University Animal Care Council, which approved all in vivo procedures and protocols. Tumor burden was calculated as the size ratio of cell-injected (left) over control (right) testicles.

### Transcriptome profiling and quantitative PCR analysis

Next-generation RNA sequencing was performed at the StemCore facility of the Ottawa Hospital Research Institute (OHRI) on an Illumina NextSeq 500 platform. Quantitative PCR analysis were performed using an ABI 7500 Real-Time PCR System. Primer sequences used in this study are presented in Table-S10.

### Bioinformatics analysis and data repository

RNA-seq alignment and specialized bioinformatics analyses were performed with the support of the Ottawa Bioinformatics Core Facility. Data were analyzed using R. For RNA-seq data, see GSE154057.

### Flow cytometry

Extracellular staining was performed using anti-CD133-PE and anti-CD44-APC (see Table-S9) and analyzed on a BD LSR Fortessa 16-color Analyzer.

### Serial organoid formation assay

Patient-derived spheroids enriched with CCSCs were dissociated, embedded in Matrigel and treated with UNC0642 (2.5 and 5 μM), BIX-01294 (1 μM), or vehicle control (DMSO) for 7 days, followed by a 7-day drug-free incubation period. For secondary passages, primary organoids were dissociated, re-plated as described above, and grown for 14 days with no further drug treatment.

### Statistical analysis

Data are presented as mean ± SEM. *P* values < 0.05 were considered significant. “n” denotes the number of times the data were replicated.

## Supplementary information

Supplementary files section

Figure S1

Figure S2

Figure S3

Figure S4

Table-S1

Table-S2

Table-S3

Table-S4

Table-S5

Table-S6

Table-S7

Table-S8

Table-S9

Table-S10

## References

[1] Jung G, Hernández-Illán E, Moreira L, Balaguer F, Goel A (2020). Epigenetics of colorectal cancer: biomarker and therapeutic potential. Nat Rev Gastroenterol Hepatol.

[2] Brenner H, Kloor M, Pox CP (2014). Colorectal cancer. Lancet.

[3] O’Brien CA, Pollett A, Gallinger S, Dick JE (2007). A human colon cancer cell capable of initiating tumour growth in immunodeficient mice. Nature.

[4] Visvader JE, Lindeman GJ (2012). Cancer stem cells: current status and evolving complexities. Cell Stem Cell.

[5] de Sousa e Melo F, Kurtova AV, Harnoss JM, Kljavin N, Hoeck JD, Hung J (2017). A distinct role for Lgr5(+) stem cells in primary and metastatic colon cancer. Nature.

[6] Malta TM, Sokolov A, Gentles AJ, Burzykowski T, Poisson L, Weinstein JN (2018). Machine Learning Identifies Stemness Features Associated with Oncogenic Dedifferentiation. Cell.

[7] Wong DJ, Liu H, Ridky TW, Cassarino D, Segal E, Chang HY (2008). Module map of stem cell genes guides creation of epithelial cancer stem cells. Cell Stem Cell.

[8] Ben-Porath I, Thomson MW, Carey VJ, Ge R, Bell GW, Regev A (2008). An embryonic stem cell-like gene expression signature in poorly differentiated aggressive human tumors. Nat Genet.

[9] Gifford CA, Ziller MJ, Gu H, Trapnell C, Donaghey J, Tsankov A (2013). Transcriptional and epigenetic dynamics during specification of human embryonic stem cells. Cell.

[10] Feldman N, Gerson A, Fang J, Li E, Zhang Y, Shinkai Y (2006). G9a-mediated irreversible epigenetic inactivation of Oct-3/4 during early embryogenesis. Nat Cell Biol.

[11] Suvà ML, Riggi N, Bernstein BE (2013). Epigenetic reprogramming in cancer. Science.

[12] Feinberg AP, Koldobskiy MA, Göndör A (2016). Epigenetic modulators, modifiers and mediators in cancer aetiology and progression. Nat Rev Genet.

[13] Wainwright EN, Scaffidi P (2017). Epigenetics and cancer stem cells: unleashing, hijacking, and restricting cellular plasticity. Trends Cancer.

[14] Kreso A, van Galen P, Pedley NM, Lima-Fernandes E, Frelin C, Davis T (2014). Self-renewal as a therapeutic target in human colorectal cancer. Nat Med.

[15] Lima-Fernandes E, Murison A, da Silva Medina T, Wang Y, Ma A, Leung C (2019). Targeting bivalency de-represses Indian Hedgehog and inhibits self-renewal of colorectal cancer-initiating cells. Nat Commun.

[16] Liu S, Ye D, Guo W, Yu W, He Y, Hu J (2015). G9a is essential for EMT-mediated metastasis and maintenance of cancer stem cell-like characters in head and neck squamous cell carcinoma. Oncotarget.

[17] Lehnertz B, Pabst C, Su L, Miller M, Liu F, Yi L (2014). The methyltransferase G9a regulates HoxA9-dependent transcription in AML. Genes Dev.

[18] Kato S, Weng QY, Insco ML, Chen KY, Muralidhar S, Pozniak J (2020). Gain-of-function genetic alterations of G9a drive oncogenesis. Cancer Discov.

[19] Pangeni RP, Yang L, Zhang K, Wang J, Li W, Guo C (2020). G9a regulates tumorigenicity and stemness through genome-wide DNA methylation reprogramming in non-small cell lung cancer. Clin Epigenet.

[20] Wang H, Cui L, Li D, Fan M, Liu Z, Liu C (2020). Overexpression of PSAT1 regulated by G9A sustains cell proliferation in colorectal cancer. Signal Transduct Target Ther.

[21] Luo CW, Wang JY, Hung WC, Peng G, Tsai YL, Chang TM (2017). G9a governs colon cancer stem cell phenotype and chemoradioresistance through PP2A-RPA axis-mediated DNA damage response. Radiother Oncol..

[22] Bergin CJ, Benoit YD (2020). G9a Is SETting the Stage for Colorectal Oncogenesis. Genes (Basel).

[23] Shi Y (2007). Histone lysine demethylases: emerging roles in development, physiology and disease. Nat Rev Genet.

[24] Benoit YD, Mitchell RR, Risueño RM, Orlando L, Tanasijevic B, Boyd AL (2017). Sam68 Allows Selective Targeting of Human Cancer Stem Cells. Cell Chem Biol.

[25] Yeung TM, Gandhi SC, Wilding JL, Muschel R, Bodmer WF (2010). Cancer stem cells from colorectal cancer-derived cell lines. Proc Natl Acad Sci USA.

[26] Sachlos E, Risueño RM, Laronde S, Shapovalova Z, Lee JH, Russell J (2012). Identification of drugs including a dopamine receptor antagonist that selectively target cancer stem cells. Cell.

[27] Werbowetski-Ogilvie TE, Bossé M, Stewart M, Schnerch A, Ramos-Mejia V, Rouleau A (2009). Characterization of human embryonic stem cells with features of neoplastic progression. Nat Biotechnol.

[28] Werbowetski-Ogilvie TE, Morrison LC, Fiebig-Comyn A, Bhatia M (2012). In vivo generation of neural tumors from neoplastic pluripotent stem cells models early human pediatric brain tumor formation. Stem Cells.

[29] Castro-Diaz N, Ecco G, Coluccio A, Kapopoulou A, Yazdanpanah B, Friedli M (2014). Evolutionally dynamic L1 regulation in embryonic stem cells. Genes Dev.

[30] Lu X, Sachs F, Ramsay L, Jacques P, Göke J, Bourque G (2014). The retrovirus HERVH is a long noncoding RNA required for human embryonic stem cell identity. Nat Struct Mol Biol.

[31] Perez CA, Ott J, Mays DJ, Pietenpol JA (2007). p63 consensus DNA-binding site: identification, analysis and application into a p63MH algorithm. Oncogene.

[32] Grade M, Hörmann P, Becker S, Hummon AB, Wangsa D, Varma S (2007). Gene expression profiling reveals a massive, aneuploidy-dependent transcriptional deregulation and distinct differences between lymph node-negative and lymph node-positive colon carcinomas. Cancer Res.

[33] Ardeljan D, Taylor MS, Ting DT, Burns KH (2017). The Human Long Interspersed Element-1 Retrotransposon: an Emerging Biomarker of Neoplasia. Clin Chem.

[34] Onder TT, Gupta PB, Mani SA, Yang J, Lander ES, Weinberg RA (2008). Loss of E-cadherin promotes metastasis via multiple downstream transcriptional pathways. Cancer Res.

[35] Benoit YD, Guezguez B, Boyd AL, Bhatia M (2014). Molecular pathways: epigenetic modulation of wnt-glycogen synthase Kinase-3 signaling to target human cancer stem cells. Clin Cancer Res.

[36] Thiery JP, Acloque H, Huang RY, Nieto MA (2009). Epithelial-mesenchymal transitions in development and disease. Cell.

[37] Luo C-W, Wang J-Y, Hung W-C, Peng G, Tsai Y-L, Chang T-M (2017). G9a governs colon cancer stem cell phenotype and chemoradioresistance through PP2A-RPA axis-mediated DNA damage response. Radiother Oncol.

[38] Tachibana M, Sugimoto K, Nozaki M, Ueda J, Ohta T, Ohki M (2002). G9a histone methyltransferase plays a dominant role in euchromatic histone H3 lysine 9 methylation and is essential for early embryogenesis. Genes Dev.

[39] Mozzetta C, Pontis J, Fritsch L, Robin P, Portoso M, Proux C (2014). The histone H3 lysine 9 methyltransferases G9a and GLP regulate polycomb repressive complex 2-mediated gene silencing. Mol Cell.

[40] Nakazawa T, Kondo T, Ma D, Niu D, Mochizuki K, Kawasaki T (2012). Global histone modification of histone H3 in colorectal cancer and its precursor lesions. Hum Pathol.

[41] Wang Z, Zang C, Rosenfeld JA, Schones DE, Barski A, Cuddapah S (2008). Combinatorial patterns of histone acetylations and methylations in the human genome. Nat Genet.

[42] Zhang T, Termanis A, Özkan B, Bao XX, Culley J, de Lima Alves F (2016). G9a/GLP Complex Maintains Imprinted DNA Methylation in Embryonic Stem Cells. Cell Rep..

[43] Roulois D, Loo Yau H, Singhania R, Wang Y, Danesh A, Shen SY (2015). DNA-Demethylating Agents Target Colorectal Cancer cells by inducing viral mimicry by endogenous transcripts. Cell.

[44] Lenstra DC, Al Temimi AHK, Mecinović J (2018). Inhibition of histone lysine methyltransferases G9a and GLP by ejection of structural Zn(II). Bioorg Med Chem Lett.

