# Supplementary material for: G9a controls pluripotent-like identity and tumor-initiating function in human colorectal cancer
Source: Oncogene. 2020 Dec 15;40(6):1191–202. doi: 10.1038/s41388-020-01591-7 (PMC7878189; doi:10.1038/s41388-020-01591-7)
Supplement: Supplementary file 1 — Supplementary files section [file 41388_2020_1591_MOESM1_ESM.docx]

**G9a controls pluripotent-like identity and tumor-initiating function in human colorectal cancer**

Christopher J. Bergin, Aïcha Zouggar, Joshua R. Haebe, Angelique N. Masibag, François M. Desrochers, Simon Y. Reilley, Gautam Agrawal and Yannick D. Benoit.

**Supplementary Files:**

**1) Extended materials and methods**

**2) Supplemental Figure legends**

**3) List of Supplemental Tables**

**4) Extended References**

**Extended materials and methods**

*Tissue culture and reagents*

Human normal intestinal epithelial crypt cells (HIEC) were cultured in OptiMEM (Gibco) supplemented with 4% FBS (Wisent Premium), HEPES, GlutaMAX (Gibco), and 10ng/ml EGF as previously described [1]. Colorectal cancer cell lines HT29, HCT116, and SW480 were obtained and cultured according recommendations from ATCC. 293FT cells for lentiviral particle production were purchased from ThermoFisher Scientific and cultured in DMEM supplemented with 10% FBS (Thermofisher), 1mM non-essential amino acids, 1mM L-Glutamine, and 1mM sodium pyruvate. Human embryonic stem cells (hESC: H9 line, WiCell) and transformed H9 ES cells (t-hESC: gift from Dr. Mickie Bhatia, McMaster University) were cultured on matrigel-coated culture plate in mTeSR media (Stemcell Technologies) according to previously established conditions [2]. All cell lines were authenticated and tested for mycoplasma. Primary colorectal tumor tissues were obtained with patient consent, as approved by the University Health Network Research Ethics Board and from Celprogen Inc. (#36112-39P, Torrance, CA). Specimen origin, tumor stage and mutational status at diagnosis are presented in Fig.S4A. Tumor samples were processed as previously described [3]. Briefly, tissues were mechanically minced and incubated with Collagenase A (3 mg/mL) for 60 min at 37°C. Following dissociation, samples were filtered using a 70μm cell strainer. Red blood cells were removed using ammonium chloride solution (Stemcell Technologies). Isolated cells were maintained as spheres in ultra-low adhesion flasks with DMEM/F-12 (Gibco) supplemented with 1% pen-strep, L-glutamine (2 mM), nonessential amino acids (1X, Gibco), sodium pyruvate (1 mM), HEPES, heparin (4 μg/mL), B27 supplement (GIBCO), N2 supplement (GIBCO), lipids mixture (Sigma), EGF (20 ng/mL) and bFGF (10 ng/mL). Small molecule inhibitors BIX-01294, UNC0642, UNC1999, and GSK LSD1 were purchased from Tocris Bioscience and re-suspended in DMSO.

*Lentiviral particles production and establishment of shRNA knockdowns*

Lentiviral knockdown vectors pLKO.1-puro shG9a (TRCN0000115670 and TRCN0000115671, Sigma) and pLKO.1-puro Non-Mammalian shRNA control (shCTRL, Sigma #SHC002) were co-transfected with packaging vectors pMD2.G and psPAX2 into 293FT cells using Lipofectamine 2000 (ThermoFisher Scientific). Viral particles were harvested as previously described [4]. Then, 5x10^5^ cells/well in 6-well plates were incubated with 0.5ml of viral suspension + 8ug/mL of polybrene (Millipore sigma) for 16 hours. Culture media containing 0.5 µg/ml of puromycin was used for 10 days of selection. Knockdown efficiency was determined by western blot.

*Cell counts and drug dose-response analysis*

Each cell model was plated at a density of 5x10^3^ cells/well in 96-well culture plates, 24h prior to drug treatments. Small molecule inhibitors BIX-01294, UNC0642, UNC1999, NCGC00244536, and GSK LSD1 were used at concentrations ranging from 0.02 to 20μM for 24 to 72 hours. Equivalent volumes of vehicle DMSO were used as control (≤0.1%). Cells were formalin-fixed, stained with Hoescht 33342, and plates were imaged with a Cellomics ArrayScan VTI High-Content imaging system (ThermoFisher Scientific). Images were analyzed HCS studio^TM^ cell analysis software and half maximum effective concentration (EC50) values were calculated using GraphPad Prism.

*Immunofluorescence*

Formaldehyde-fixed paraffin-embedded human colon carcinoma tissue microarray sections, including normal colon tissues (US Biomax, #CO486) were rehydrated, quenched in 0.1M glycine buffer, and blocked with a 2.5% BSA-PBS solution prior to immunostaining, as previously described [5]. For staining of cells, culture wells were fixed with 2% formalin and incubated in Perm/Wash buffer (BD Biosciences) at 4^o^C for 15-30 min prior to immunostaining [4]. Anti-H3K9me2 primary antibody (Table-S9) was diluted at 1:500 in 1% BSA-PBS solution and incubated overnight at 4°C. Secondary antibody (anti-mouse Alexa fluora 488) was used at 1:500 in a 1% BSA-PBS solution. For staining on tissue sections, slides were mounted using Vectashield mounting medium with DAPI (Vector labs). For cell staining, nuclei were stained with Hoechst 33342. Cells and tissue sections were imaged with a Cellomics ArrayScan VTI High-Content imaging system and fluorescence quantification was performed with HCS studio^TM^ cell analysis software for cell-based assays and Image J software (National Institutes of Health) for tissue sections.

*Western blotting*

Whole-cell extracts were prepared in Laemmli Sample Buffer (60mM Tris-HCL pH 6.8, 2% SDS, 10% glycerol, 5% β-mercaptoethanol, 0.01% bromophenol blue), sonicated and thermo-reduced/denatured (5 min, 95^o^C) prior to electrophoresis on 12% or 15% polyacrylamide gels. Proteins were transferred onto nitrocellulose membranes and immuno-detected as previously described [4]. For dot blot experiments, whole-cell extracts were directly spotted onto membranes and air-dried for 30 min prior to blocking. Membranes were blocked in PBS containing 5% skim milk and 0.1% TWEEN 20. Primary and secondary antibodies used are described in Table-S9. Full-range molecular mass marker (Full-Range Rainbow Marker, VWR) was used as standard. Blot images were acquired using a ChemiDoc MP Imaging system. Quantitative optical densitometry analysis of bands was performed using Image J software (National Institutes of Health).

*In vivo teratoma assays*

Stable control and G9a-knockdown t-hESCs were treated with collagenase IV and harvested using TrypLE dissociation reagent (ThermoFisher) and injected intratesticularly into 12-week old male NOD/SCID mice. Specifically, 5x10^5^ t-hESC were injected into the left testicle of each mouse, as previously described [2]. Right testicles were injected with an equivalent volume of saline solution, as a "no-cell" injection control. At 14-day post-injection, mice were euthanized and testicles size was measured for each animal using a caliper. Tumor burden was calculated as the size ratio of cell-injected (left) over control (right) testicles. Volume of each testicle was calculated as 1/2 x length x width^2^.

*Methylated DNA Immunoprecipitation (MeDIP) assay*

MeDIP experiments were performed using the EpiMark Methylated DNA Enrichment Kit (New England Biolabs), according to the manufacturer’s instructions. t-hESCs were treated with BIX-01294 or UNC0642 for 48h vs. equivalent volume of control DMSO. Genomic DNA (gDNA) was extracted using the DNeasy Blood & Tissue kit (Qiagen). Following MeDIP procedure, input and output fractions were analyzed by qPCR. Input gDNA quantification was done via MirA amplification (specific primers included in the kit). HeLa control DNA as well as specific primers to amplify total LINE elements and low-methylated control region RPL30 were also provided in the kit. Sequences of primers designed to detect L1-ORF2 L1-5’UTR, and hERV-H genomic elements were presented in Table-S10, and used as previously reported[6].

*Transcriptome profiling and quantitative PCR analysis*

RNA was extracted using the total RNA purification kit by Norgen Biotech Corp, following the manufacturer's guidelines. Quantification of total RNA samples was performed with a Qubit HS RNA assay (ThermoFisher Scientific) and fragment size was evaluated with a Fragment Analyzer HS NGS assay (AATI). A RNA Quality Number (RQN) of 8.0 or higher was considered satisfactory for library construction. Library construction was performed with a Truseq RNA v2 (Illumina). Libraries were prepared with unique barcodes compatible with the Illumina NextSeq 500 platform. Quantification of the libraries was performed with a Qubit HS DNA assay and library fragment size was evaluated with a Fragment Analyzer HS NGS assay. Libraries were normalized to the same concentration, then samples were pooled in equal amounts. Next-generation RNA sequencing was performed on an Illumina NextSeq 500 platform, according to 1 x 75bp cycles of single-end sequencing, yielding 25 million reads per sample. PhiX ssDNA was spiked in each sample and used as a technical control for clustering reactions. Upon alignment of sequencing data, control vs. treated, transcriptomes for each cell line were compared and significantly modulated genes (p<0.05) were identified using the Salmon transcript abundance method [7]. For quantitative PCR analysis, cDNA was synthesized from 1μg of total RNA using SuperScript VILO cDNA Synthesis Kit (Invitrogen). qPCR reactions were carried out using Power SYBR Green Master Mix (ThermoFisher Scientific) per manufacturer’s recommendation. Amplification was performed using an ABI 7500 Real-Time PCR System. Primer sequences used in this study are presented in Table-S10. Primers for L1-ORF1 and L1-5’UTR transcripts were used as previously reported[8]. All reactions were normalized to GAPDH as reference gene, and relative gene quantification was calibrated against vehicle/control-treated samples according to a method previously described [9].

*Survival analysis*

Hazard ratios relative to the expression chromatin modifiers based on disease free survival were calculated using the platform GEPIA2 [10]. Samples within the 85th percentile for expression of each chromatin regulators, determined by RNA-seq in TCGA cohorts were ranked as high-expressing tumors. Statistical significance cutoff was set at p<0.05.

*Bioinformatics analysis and data repository*

RNA-seq alignment and specialized bioinformatics analyses were performed with the support of the Ottawa Bioinformatics Core Facility. Data was analyzed using R. (Tags Per Million) values genes and patients of interest were downloaded from TCGA using TCGAbiolinks [11]. TPM values were multiplied by 1 million for ease of interpretation in spreadsheets. Clustering and heatmap visualization was performed using heatmap.2. Gene expression data for cell lines were presented as fold-change (Salmon transcript abundance method) or as log transformed row-mean normalized FPKM/RPKM. Data for HCT116 transcriptome profiling is from GSE61255 data set (control: GSM1500847, and BIX-01294: GSM1500848). Stem cell index calculation was performed as previously described [12]. GSEA analysis were performed as a pre-ranked analysis using GSEA software version 4.0.3 (www.broadinstitute.org/gsea) with 1000 permutations[13]. Normalized enrichment scores ± 1.5 and p<0.05 were considered significant. Information on gene signatures used in GSEAs is presented in Table-S8. Gene ontology analysis was performed using the ICGC Data Portal (https://dcc.icgc.org/) where enrichment analysis was preformed against GO Molecular Function, GO Biological Process and GO Cellular Component with an FDR q-value threshold of 0.05. ChIP-seq reads from GSE82131 data set were quantified in 1kb bins genome-wide for G9a and H3K9me2. Bins were filtered against the ENCODE blacklist and mitochondria as well as unmapped contig assemblies were excluded. Top 20K signal/bins for both G9a and H3K9me2 were overlapped, yielding 1,392 co-enriched regions. Nearest gene annotation was performed using the R ChIPseeker package. A list of 968 genes associated with ≥1 bin(s) is presented in Table-S4. Log2 fold change expression values for genes co-occupied by G9a/H3K9me2 in CCSCs, and significantly modulated in HT29 vs. HIEC cells are presented in Table-S6. The accession number for the RNA-seq data newly reported in this paper is: GSE154057. Dendrograms of hierarchical gene set clustering were generated using Morpheus (https://software.broadinstitute.org/morpheus/).

*Flow cytometry*

Extracellular staining was performed in PBS supplemented with 3% FBS and 0.5mM EDTA (PEF) where 100K/ml cells of interest were incubated with antibodies for 1hour at 4°C, washed with 10 volumes of PEF and then stained with 7-amino actinomycin D (7-AAD, Immunotech) to eliminate dead cells prior to analysis. Single-cell suspensions were stained using anti-CD133-PE and anti-CD44-APC (see Table-S9). Flow cytometry analysis was performed on a BD LSR Fortessa 16-colour Analyzer and data analysis was conducted using FlowJo (Tree Star Inc)[4].

*Serial organoid formation assay*

Patient-derived spheres enriched with CCSCs were harvested and dissociated using TrypLE reagent (ThermoFisher) and passed through a 70-μm strainer to eliminate non-single-cell aggregates. Cell suspensions were mixed with Matrigel in sphere culture media (1:1 ratio) to get a 1-cell/μl density. Mixtures were immediately plated as 300 μl domes in 6-well plates and incubated for 15 minutes at 37°C for Matrigel polymerization. Then, 2.5mL of sphere media containing doses of UNC0642 (2.5 and 5μM), BIX-01294 (1μM), or vehicle control (DMSO) was added to each well. Drug treatment lasted for 7 days, followed by a 7-day drug-free incubation period. At day-14, plates were imaged using a Cellomics ArrayScan VTI platform to determine organoid counts and size using HCS Studio^TM^ software. For secondary passage experiments, control primary organoids and those remaining in treated wells were dissociated using a gentle dissociation reagent (STEMCELL Technologies) and re-plated as described above, but according to a 50-cell/μl density. Secondary organoids were grown for 14 days in sphere culture media with no further drug treatment. Then, secondary organoid counts were determined using a Cellomics ArrayScan VTI platform and HCS Studio^TM^ software. For organoid assays involving HIEC cells, monolayer cultures were treated with NCGC00244536 (5μM), over 48 hours (vs. DMSO controls), prior to a 24-hour recovery time. Next, HIEC cells were dissociated, mixed with Matrigel in OptiMEM 4% FBS 10ng/ml EGF media, at a 1-cell/μl density, and plated as 300 μl domes in 6-well plates. HIEC organoid were analyzed as described above.

*Statistical analysis*

Data is presented as mean ± SEM. P-values < 0.05 were considered significant. ‘‘n’’ denotes the number of times the data was replicated. Significant differences between groups were determined by 2-way ANOVA test and unpaired two-tailed Student t-test, using GraphPad Prism software, except for differentiation marker assessment (Fig.3F), where one-tail Student t-test was applied (unidirectional analysis). All tests assume Gaussian distribution and equal SD.

**Supplemental Figure Legends**

Figure-S1:

**A)** Kaplan-Meier disease-free survival analysis of patients presenting high (top 15%, TPM+1) and low (bottom 85%, TPM+1) expression levels of DNMT3A, KDM4B, and EHMT1/GLP in primary CRC tumors (TCGA COAD, n=275, Logrank p<0.05 is significant).

**B)** Box plots of key H3K9me2 modulators profiled by RNA-seq in human colon adenocarcinoma (TCGA COAD, n=275 vs. normal, n=41). Values are expressed as TPM (***: p<0.0001, Log2FC cutoff >0.5).

**C)** Quantitative PCR analysis of G9a and LSD1 expression in normal HIEC, SW480, HCT116, HT29 CRC lines, as t-hESCs. GAPDH was used as a reference gene (n=6, ***: p≤0.001; **: p≤0.007; *: p=0.027).

Figure-S2:

**A)** Immunofluorescence detection of H3K9me2 mark in non-silencing shCTRL and shG9a t-hESCs. Nuclei were counterstained with DAPI (20X magnification).

**B)** Phase-contrast micrographs of shCTRL and shG9a t-hESCs compared to normal human ES cells (hESC) (10X magnification). Arrowheads are marking the presence of sharp colony edges.

**C)** Western blot analysis of H3K27me1 and H3K27me3 levels in BIX-01294 (1μM, 48h) and UNC0642 (5μM, 48h) t-hESCs vs. DMSO controls. Total Histone H3 and GAPDH were used as loading controls. Relative OD signal quantification vs. GAPDH intensity is presented in the bar graph.

**D)** MeDIP assay performed on control HeLa DNA showing specific enrichment of methylated DNA from LINE elements vs. low-methylated region RPL30 (*: p=0.016). Analysis of flow through fractions (unbound) show lower abundance of LINE elements vs. low-methylated RPL30 (*: p=0.022).

**E)** MeDIP experiments assessing methylation levels in t-hESCs LINE transposable elements (total LINEs, LINE1 5’-UTR), endogenous retroviral elements hERV-H, and control RPL30 upon BIX-01294 (1μM, 48h) and UNC0642 (5μM, 48h) treatments (vs. DMSO). Methylated DNA values for each pulled down region is presented as relative to input.

**F)** Expression of transcripts encoding endogenous retroviral (hERVs) and LINE1 elements in t-hESCs (BIX-01294 1μM vs. DMSO) and HIEC cells. hERVs mRNA levels were determined by RNA-seq (heat map) while LINE1 expression was measured by qPCR (n=3, t-hESC BIX vs. DMSO: ***: p=0.00005; **: p=0.0035).

**G)** Volcano plot displaying differentially expressed genes in BIX-01294 (1μM, 48h) treated vs. DMSO. Statistically significant genes (p<0.05) with <5% FDR value (highlighted in red) were used in subsequent experiments.

**H)** Relative mRNA expression of core pluripotency factors in t-hESCs treated with BIX-01294 (1μM, 48h) or vehicle control (DMSO) determined by RNA-seq profiling.

**I)** GSEA showing a positive correlation between BIX-01294-induced transcriptional changes (vs. DMSO, p<0.05) in t-hESCs and p53 target genes (M4391).

**J)** Candidate-based validation of Cell cycle and Differentiation modulator genes upon G9a inhibition using UNC0642 (5μM, 48h) in t-hESCs (n=3, **: p=0.0074; *: p≤0.026).

Figure-S3:

**A)** Heat maps representing RNA-seq profiling of genes upregulated in ES cells and p53 targets in HCT116 human CRC line upon BIX-01294 treatment (GSM1500848) vs. vehicle control (GSM1500847). ES cells genes and p53 targets modulated by BIX-01294 in similar directions in HCT116 vs. t-hESCs were marked with asterisks. # marks genes modulated in opposite directions. Bar graphs illustrate the number of up/down-regulated genes in BIX-01294-treated HCT116 vs. vehicle control.

**B)** Candidate-based validation of genes from WONG Embryonic Stem Cell Core (M7079) and PEREZ TP53 Targets (M4391) lists upon G9a inhibition using UNC0642 (2.5μM, 48h) in HCT116 cells (n≥3, ***: p≤0.0008; **: p=0.0025; *: p≤0.017).

**C)** Western analysis of H3K9me2 levels in shCTRL vs. shG9a and BIX-01294 (1μM, 48h), UNC0642 (2.5μM, 48h) and DMSO-treated HT29 cells. Actin was used as loading control. Relative OD signal quantification for H3K9me2 vs. Actin intensity is presented.

**D)** Western blot analysis of H3K9me2 levels upon pharmacological inhibition of KDM4B demethylase activity using NCGC00244536 (5μM, 48h) in HIEC and HT29 cells. Histone H3 and Actin were used as loading controls. Relative OD signal quantification vs. Actin intensity is presented in the bar graph (n=4, **: p=0.0016).

**E)** Dose-response curves assessing toxicity of KDM4B inhibition (NCGC00244536) in CRC stem-like HT29 vs. normal HIEC cells.

**F)** Validation of G9a knockdown efficiency in HT29 cells using shRNA clones #TRCN0000115670 (shG9a #1) and # TRCN0000115671 (shG9a #2) vs. scramble control shRNA #SHC002 (shCTRL).

Figure-S4:

**A)** Table of clinical information for CRC patients involved in this study.

**B)** Representative micrographs of primary organoids from 2.5μM and 5μM UNC0642-treated vs. DMSO control groups (4X magnification). Average organoid size quantification is presented for the same experimental conditions (n=11, ***: p≤0.0006).

**C)** Primary and secondary organoid formation frequency observed upon BIX-01294 treatments (1μM) vs. DMSO controls (2 patients: #92 and #146, n≥3, ***: p=0.0001), as well as H3K9me2^High^ vs. H3K9me2^Low^ HIEC cells (n=3).

**D)** Patient-specific response in secondary organoid formation assays for UNC0642-treated primary samples vs. DMSO controls (**: p=0.0065, *: p≤0.0171).

**E)** Read counts for 1Kb regions detected by G9a and H3K9me2 ChIP-seq. 1Kb regions were filtered against the ENCODE blacklist. Left panel represents chromosome, unmapped assemblies, and mitochondria regions. Right panel represents only chromosome-mapped 1Kb regions, which were used for subsequent analyses.

**F)** Pie chart summarizing genomic distribution of retained 1392 co-occupied 1Kb regions. Nearest gene annotation for each 1Kb region is presented in Table-S4.

**G)** Volcano plot displaying differentially expressed genes between HT29 and HIEC cells. Statistically significant genes (p<0.05) with <5% FDR value are highlighted in red and were used in related analyses.

**H)** Heat map representing RNA-seq profiling of genes involved in cell differentiation (GO_Cell_Differentiation: GO:0030154), co-enriched with G9a and H3K9me2 in human CCSCs (GSE82131), and silenced in HT29 vs. HIEC cells (p<0.05). Relative mRNA levels of G9a in HT29 vs. HIEC is also presented (n=3, ***: p<0.0001).

**List of Supplemental Tables**

Table-S1: List of 121 COAD patient samples used in chromatin regulators expression analysis

Table-S2: Expression of chromatin regulators in High and Low mRNAsi COAD samples

Table-S3: List of significantly modulated genes in BIX-treated t-hESCs vs. vehicle

Table-S4: Annotation of regions co-occupied by G9a and H3K9me2 in CCSCs from top-20,000 1Kb bins with most read counts

Table-S5: Genes significantly downregulated in HT29 vs. HIEC (Top-100)

Table-S6: Genes co-occupied by G9a and H3K9me2 in CCSCs and significantly modulated in CRC vs. normal intestinal progenitor cells

Table-S7: GO_terms highlighted in gene ontology analyses

Table-S8: Information on gene signatures used in GSEAs

Table-S9: List of primary antibodies used in this study

Table-S10: List of qPCR primers used in this study

**Extended References**

1 Perreault N, Beaulieu JF. Use of the dissociating enzyme thermolysin to generate viable human normal intestinal epithelial cell cultures. *Exp Cell Res* 1996; 224: 354-364.

2 Werbowetski-Ogilvie TE, Bossé M, Stewart M, Schnerch A, Ramos-Mejia V, Rouleau A *et al*. Characterization of human embryonic stem cells with features of neoplastic progression. *Nat Biotechnol* 2009; 27: 91-97.

3 Kreso A, O'Brien CA. Colon cancer stem cells. *Curr Protoc Stem Cell Biol* 2008; Chapter 3: Unit 3.1.

4 Benoit YD, Mitchell RR, Risueño RM, Orlando L, Tanasijevic B, Boyd AL *et al*. Sam68 Allows Selective Targeting of Human Cancer Stem Cells. *Cell Chem Biol* 2017; 24: 833-844.e839.

5 Benoit YD, Pare F, Francoeur C, Jean D, Tremblay E, Boudreau F *et al*. Cooperation between HNF-1alpha, Cdx2, and GATA-4 in initiating an enterocytic differentiation program in a normal human intestinal epithelial progenitor cell line. *Am J Physiol Gastrointest Liver Physiol* 2010; 298: G504-517.

6 Muotri AR, Marchetto MC, Coufal NG, Oefner R, Yeo G, Nakashima K *et al*. L1 retrotransposition in neurons is modulated by MeCP2. *Nature* 2010; 468: 443-446.

7 Patro R, Duggal G, Love MI, Irizarry RA, Kingsford C. Salmon provides fast and bias-aware quantification of transcript expression. *Nat Methods* 2017; 14: 417-419.

8 Guo H, Chitiprolu M, Gagnon D, Meng L, Perez-Iratxeta C, Lagace D *et al*. Autophagy supports genomic stability by degrading retrotransposon RNA. *Nat Commun* 2014; 5: 5276.

9 Pfaffl MW. A new mathematical model for relative quantification in real-time RT-PCR. *Nucleic Acids Res* 2001; 29: e45.

10 Tang Z, Kang B, Li C, Chen T, Zhang Z. GEPIA2: an enhanced web server for large-scale expression profiling and interactive analysis. *Nucleic Acids Res* 2019; 47: W556-W560.

11 Colaprico A, Silva TC, Olsen C, Garofano L, Cava C, Garolini D *et al*. TCGAbiolinks: an R/Bioconductor package for integrative analysis of TCGA data. *Nucleic Acids Res* 2016; 44: e71.

12 Malta TM, Sokolov A, Gentles AJ, Burzykowski T, Poisson L, Weinstein JN *et al*. Machine Learning Identifies Stemness Features Associated with Oncogenic Dedifferentiation. *Cell* 2018; 173: 338-354.e315.

13 Subramanian A, Tamayo P, Mootha VK, Mukherjee S, Ebert BL, Gillette MA *et al*. Gene set enrichment analysis: a knowledge-based approach for interpreting genome-wide expression profiles. *Proc Natl Acad Sci U S A* 2005; 102: 15545-15550.
