# Supplementary figures and images for: G9a controls pluripotent-like identity and tumor-initiating function in human colorectal cancer
Source: Oncogene. 2020 Dec 15;40(6):1191–202. doi: 10.1038/s41388-020-01591-7 (PMC7878189; doi:10.1038/s41388-020-01591-7)

A

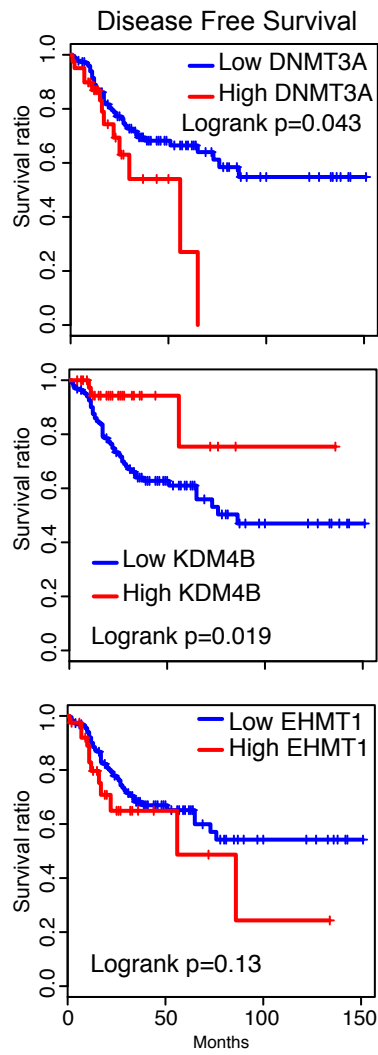

B

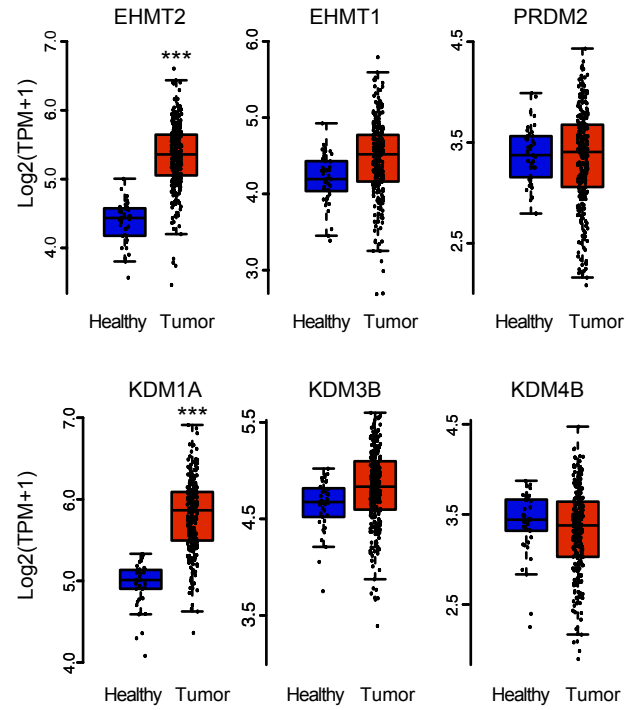

C

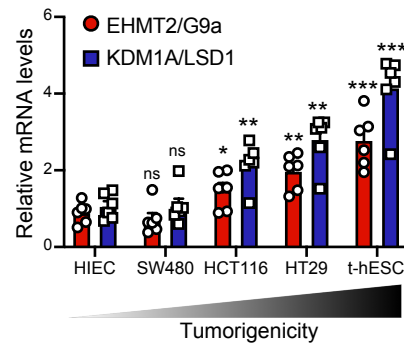

Supplement: Supplementary file 2 — Figure S1 [file 41388_2020_1591_MOESM2_ESM.pdf]

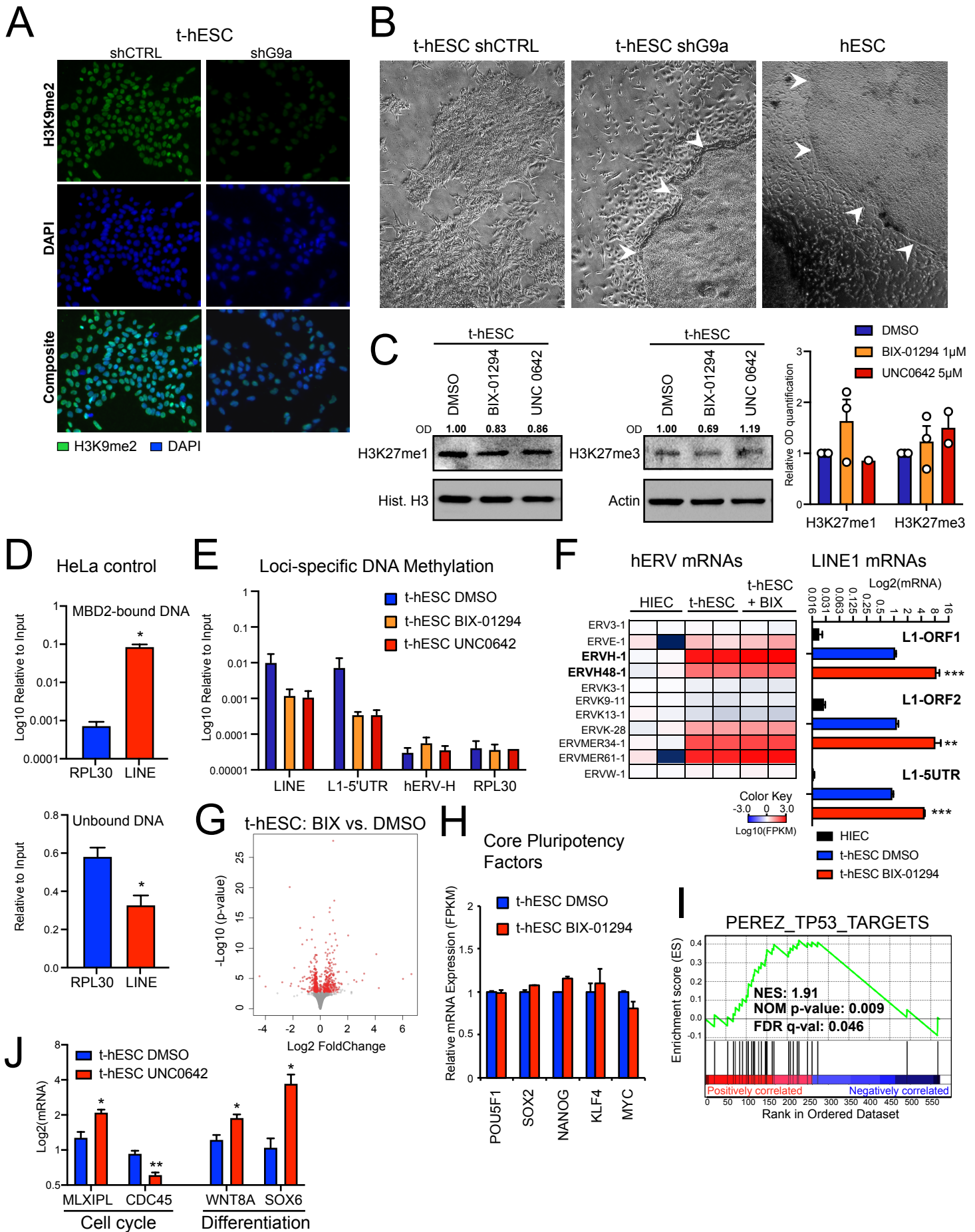

Supplement: Supplementary file 3 — Figure S2 [file 41388_2020_1591_MOESM3_ESM.pdf]

A

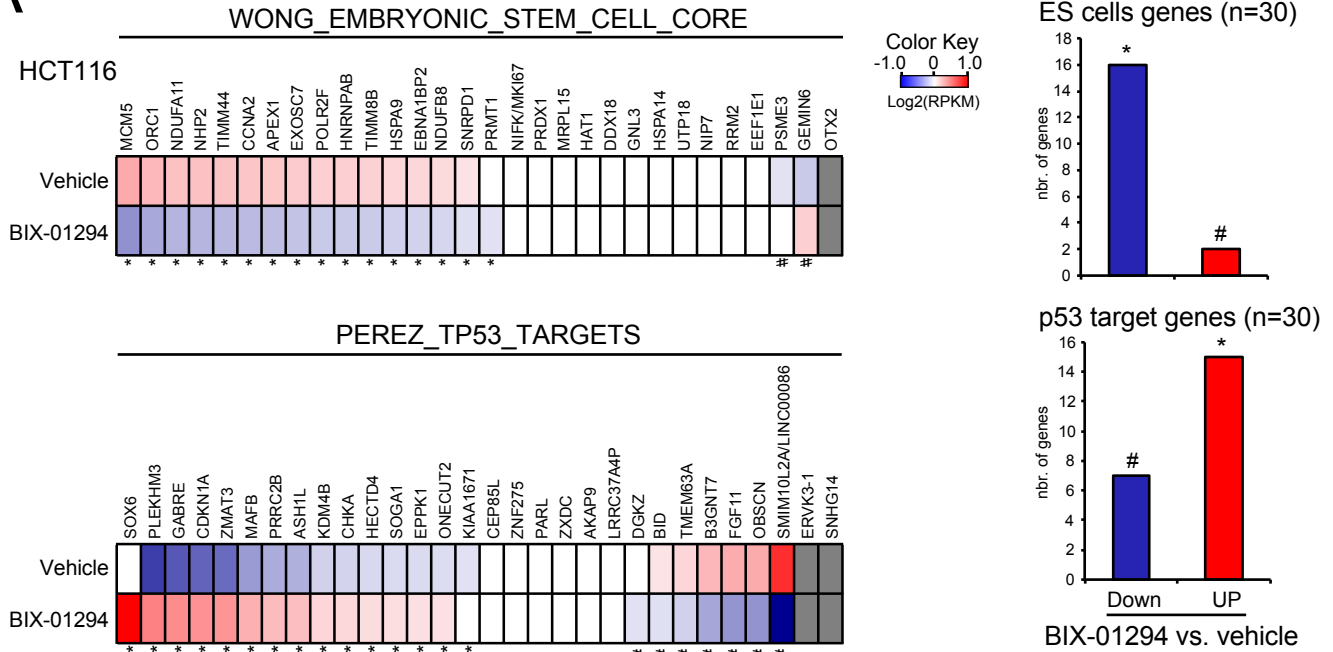

B

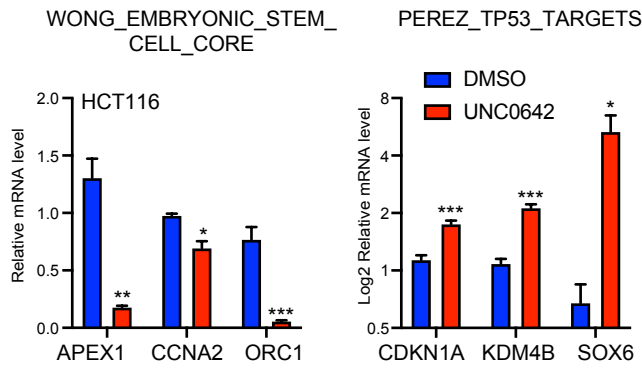

C

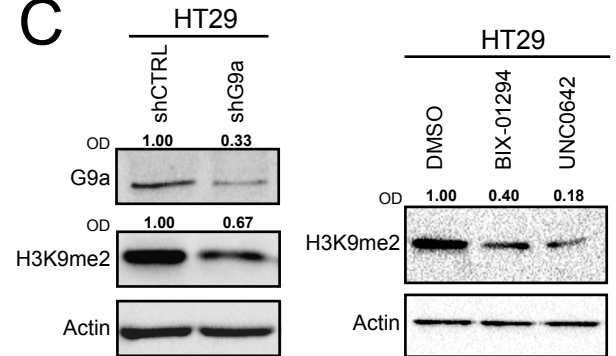

D

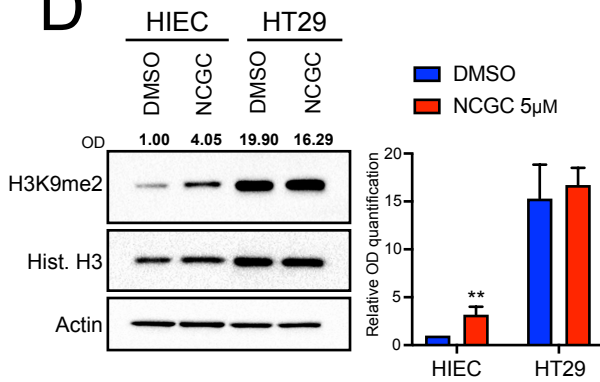

E

Normal vs. Transformed:  
KDM4B inhibition

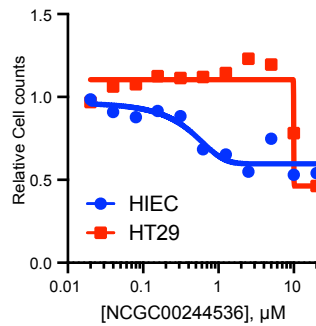

F

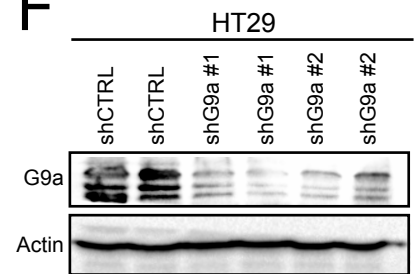

Supplement: Supplementary file 4 — Figure S3 [file 41388_2020_1591_MOESM4_ESM.pdf]

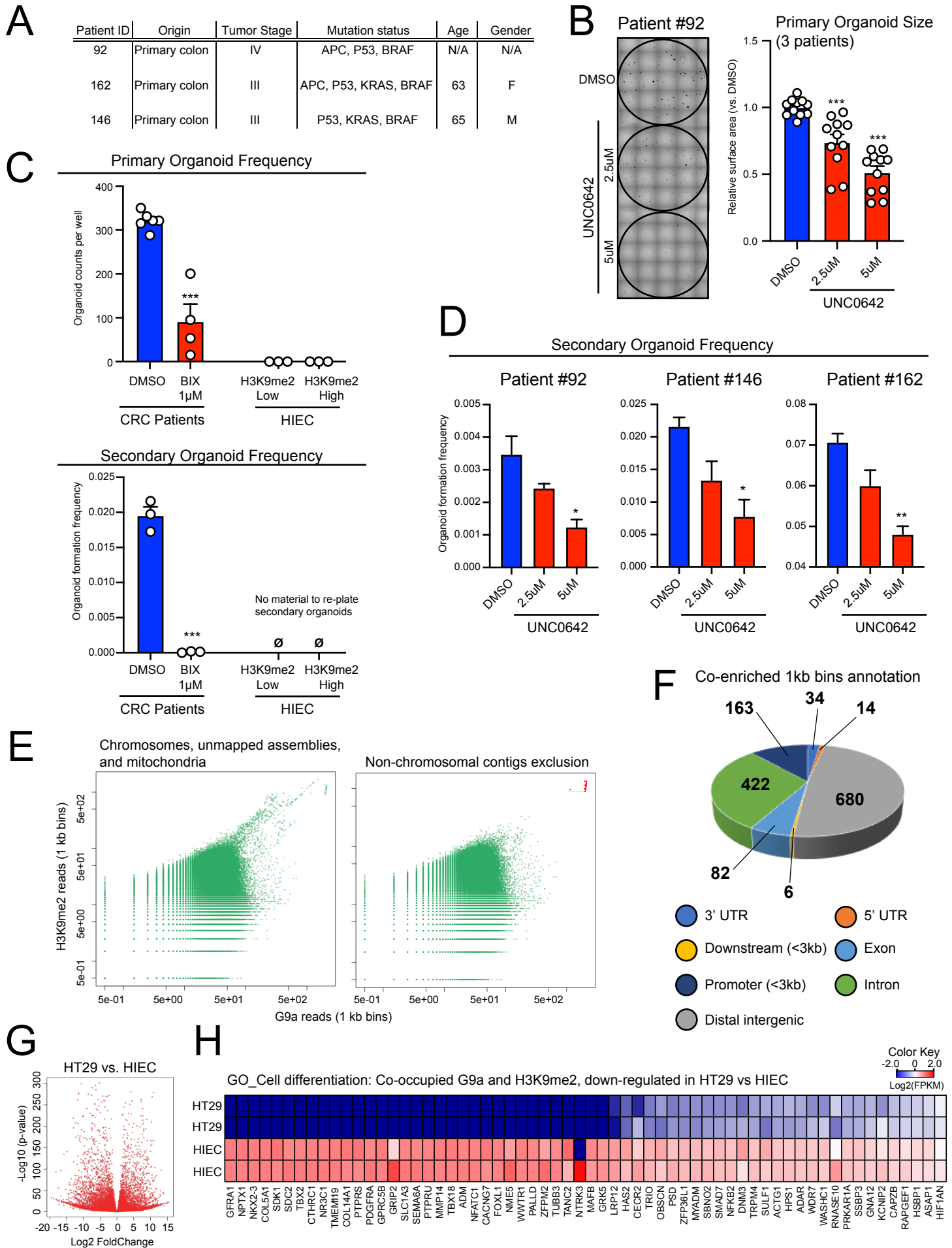

Supplement: Supplementary file 5 — Figure S4 [file 41388_2020_1591_MOESM5_ESM.pdf]
